# Supplementary material for: Temporal propagation of neural state boundaries in naturalistic context
Source: Cereb Cortex. 2025 Oct 17;35(10):bhaf284. doi: 10.1093/cercor/bhaf284 (PMC12533692; doi:10.1093/cercor/bhaf284)
Supplement: Supplementaries_bhaf284 [file supplementaries_bhaf284.pdf]

Temporal Propagation of Neural State Boundaries in Naturalistic Context

Djamari Oetringer[1,*], Sarah Henderson[2], Dora Gözükara[1], Linda Geerligs[1]

[1] *Donders Institute for Brain, Cognition and Behaviour, Radboud University, Nijmegen, the Netherlands*
[2] *Department of Psychology, University of Texas, Austin, Texas, United States*

Supplementary materials

[*] Corresponding author:
djamari.oetringer@donders.ru.nl
Thomas Van Aquinostraat 4, 6525GD,Nijmegen, the Netherlands

A Artifacts in data

After excluding subjects based on age, language hemisphere, and brain anatomy, we visually inspected the timeseries of all electrodes and the time by time correlations of all remaining subjects. As all subjects were patients with severe epilepsy, more atypical brain activity can be expected as compared to healthy controls. Based on these visualizations, another 5 subjects were excluded due to the presence of atypical signals, which we classified as artifacts from unknown sources. For comparison, the data of two included subjects are visualized in Figure S1.

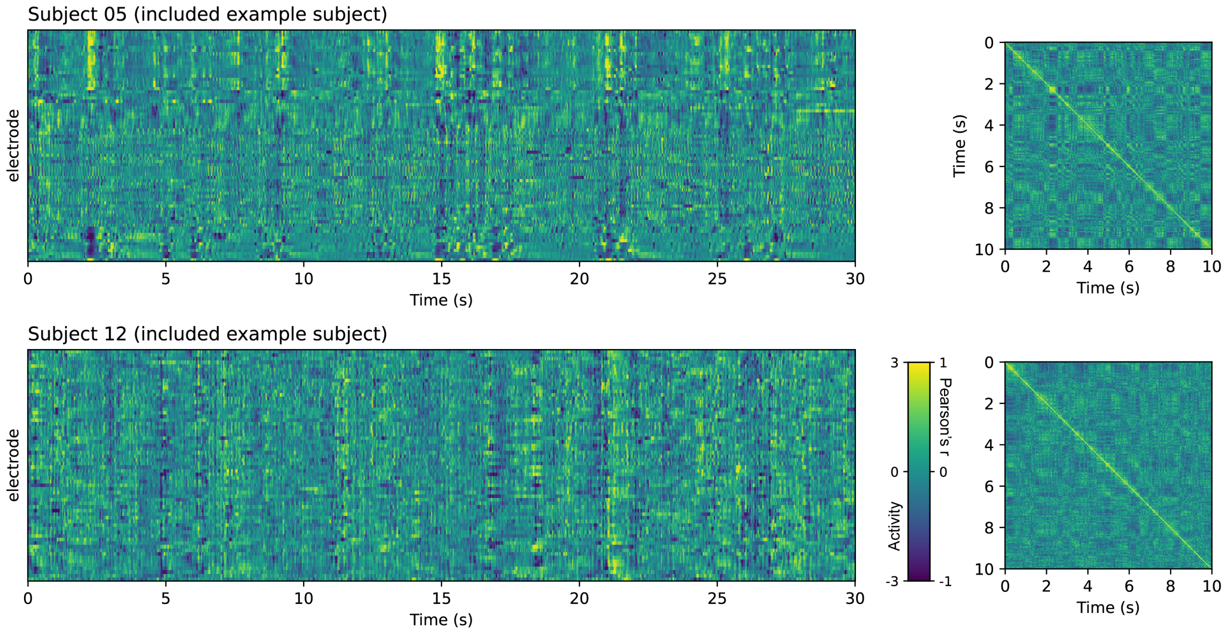

Fig. S1. Preprocessed data of one example block for two subjects that were included in the final analyses. Left: timeseries of a full 30-second block. Right: time by time correlation matrices of the first 10 seconds of that block.

For each subject that was excluded on the basis of visual inspection, the preprocessed data of one block of 30 seconds in visualized in Figure S2. Note that the data in this figure was re-referenced using the ICA-based method, but for every depicted subject the artifacts were also present when using the common average re-reference.

Subject 03 and 07

Subjects 03 and 07 had a rhythmic artifact throughout the whole stimulus, although the rhythm of subject 07 seems to have a higher frequency. The presence of such a rhythm is especially apparent in their time by time correlations, which form a pattern similar to a checkerboard. The durations of the periods with relatively high correlations are more consistent than what can be expected from typical neural activity.

Subject 48 and 57

Both subject 48 and 57 had bursts of atypical signal with periods of very high or very low neural activity. This was especially apparent in their timeseries but also reflected in their time by time correlations. This can be seen once in each example block depicted in the figure, but also occurred at other moments throughout the whole stimulus.

Subject 60

Many electrodes of subject 60 had non-synchronized atypical activity, which can be seen as wide horizontal stripes of prolonged negative or positive activity in the timeseries. The set of electrodes with such atypical activity differed per block, though there was some overlap. Given the high number of bad electrodes, especially when taking all blocks together, we decided to exclude this subject.

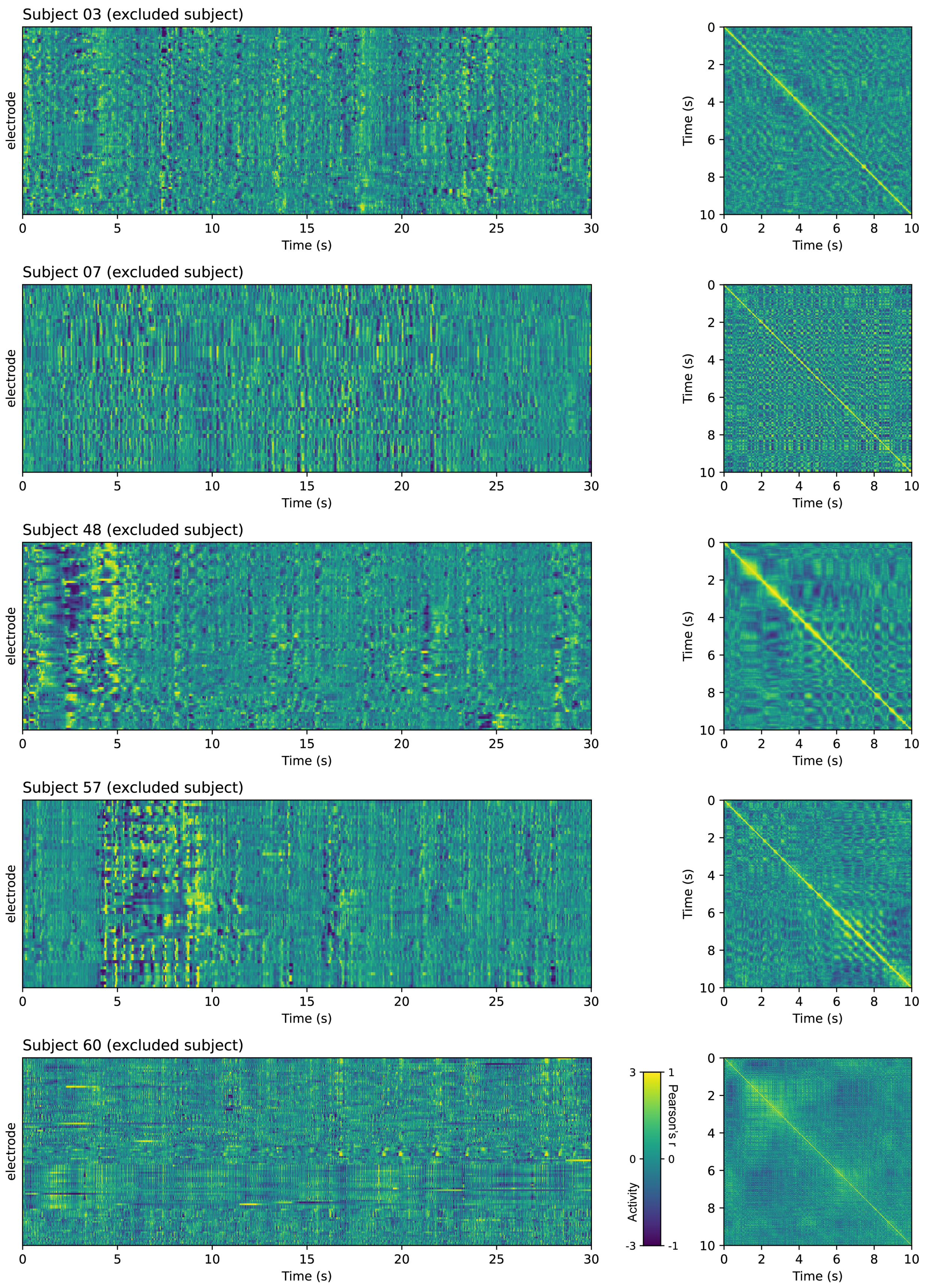

Fig. S2. Preprocessed data of one example block for each subject that was excluded from the analyses based on visual inspection of their data. Left: timeseries of the full 30-second block. Right: time by time correlation matrices of the first 10 seconds of that block.

B Subject descriptions

The number of electrodes used in the analyses per subject per ROI can be found in the table below. Note that subject number 12 had no electrodes in the low-level ROI and was therefore excluded from any of the analyses that required this ROI. 6 subjects viewed the stimulus only once ("Novel"), while 5 subjects ("Familiar") had seen the stimulus before.

Subject number	Group	Number of electrodes in low-level ROI	Number of electrodes in high-level ROI
**05**	Novel	30	24
**12**	Novel	0	18
**22**	Familiar	12	15
**26**	Novel	26	12
**36**	Novel	32	21
**45**	Familiar	21	23
**46**	Familiar	31	21
**51**	Familiar	17	13
**54**	Novel	24	29
**55**	Familiar	26	22
**59**	Novel	21	21

Table S1. Summary descriptions of the used electrodes per subject.

C Example timelines of neural state boundaries

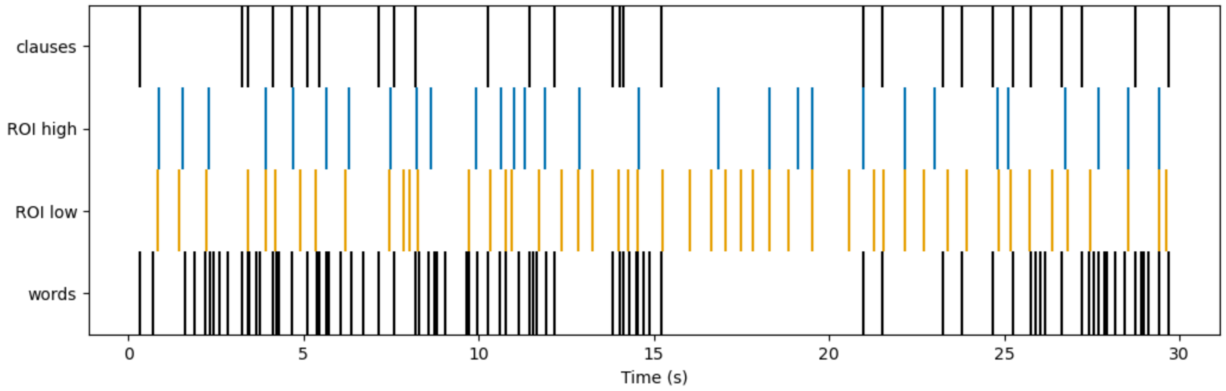

Fig. S3. Timeline of the onsets and offsets of clauses and words of one example block, together with the neural state boundaries in the low- and high-level ROI of one example subject. No delay has been applied.

The timings of annotation boundaries of an example run are visualized in Figure S3, together with the neural state boundaries in both the low-level and high-level ROI of one example subject.

D Duration hierarchy using smaller high-level ROIs

When investigating the neural state durations in our main analysis, we expected the high-level ROI to have longer states than the low-level ROI. However, the results were inconsistent across subjects with 6 subjects having longer neural states in the low-level ROI, and 4 subjects having longer neural states in

the high-level ROI. Given that the high-level ROI consists of multiple anatomically separated Brodmann areas, here we studied whether applying GSBS and subsequent analysis on smaller and anatomically connected areas would give rise to more consistent results. Therefore, we divided the Brodmann areas up into 3 smaller areas: temporal pole (TP; Brodmann area 38), angular gyrus and connected areas (AG+; Brodmann areas 39 and 40), and Broca and connected areas (Broca+; Brodmann areas 44, 45, 46, and 47). We re-ran GSBS and subsequent analyses per subject only if the number of electrodes in a smaller area was at least 10. If the duration hierarchy was indeed inconsistent in the main analysis because of these anatomically separated areas being taken together, then we expect all of these smaller areas to show longer median durations as compared to the low-level ROI in the same subject. This was however not the case for any of the three areas (Figure S4). TP had the exact same median state duration as the low-level ROI in 1 subject, and shorter states in TP than the low-level ROI in another subject. AG+ had longer states than the low-level ROI in 2 subjects, and shorter states in another 2 subjects. Finally, Broca+ had longer states than the low-level ROI in 2 subjects, and shorter states in another 2 subjects. Taking everything together, we conclude that extracting neural states from anatomically connected Brodmann areas only still does not give rise to the neural state duration hierarchy found in previous fMRI studies. Together with the results of our main analysis, we conclude that the neural state duration hierarchy is absent in these data.

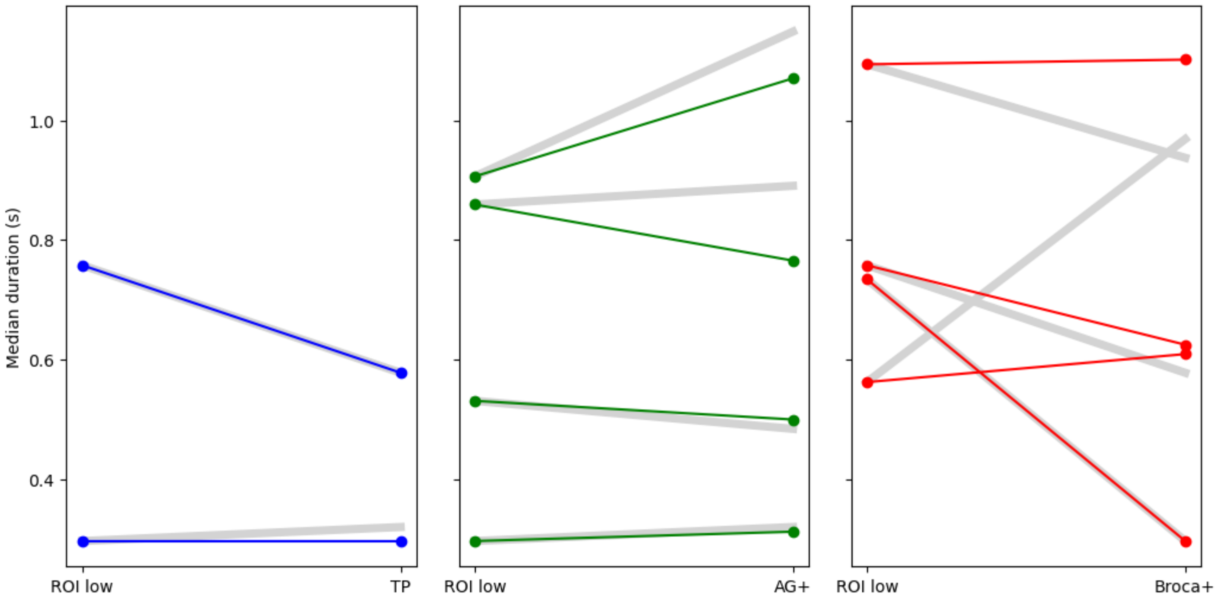

Fig. S4. Median state duration in the low-level ROI compared to the high-level ROI, but divided up into three smaller high-level areas: temporal pole (TP), angular gyrus and connected areas (AG+), and Broca and connected areas (Broca+). Each colored line is one subject, with the gray thick line indicating the median durations in the low-level ROI compared to the high-level ROI that was used in the main analysis.

E Anticipation analysis

To ensure that having seen the stimulus before did not impact the results, we investigated the timing differences of neural state boundaries of subjects who had already seen the stimulus (the Familiar group) to those of the subjects who viewed the stimulus for the first time (the Novel group). More specifically, we tested whether boundaries of the Familiar group occurred earlier in time than those of the Novel group. Per subject pair, we first selected a group of electrodes that the subjects had "in common". Here,

“in common” means that the electrodes were located in the same area according to the Brainnetome Atlas (Fan et al., 2016). If one subject had more electrodes in a particular area than in another, random electrodes of that subject were dropped until the number of electrodes within a particular area was equal between the two subjects. We then ran GSBS on both subjects separately, using all selected electrodes across the brain as input, and computed the optimal delay with clauses as was done in our main analysis. GSBS and consequently the optimal delay was only computed if the number of electrodes that the two subjects had in common was at least 15. By doing this for all subject pairs, we created a subject by subject matrix indicating the difference in optimal delay with clauses for each subject pair (Figure S5A). The measurement to be statistically tested was the median of the lower-left quadrant, which indicated how much later the optimal delay of the familiar subjects was compared to the novel subjects. Based on (Lee et al., 2021), this number is expected to be negative as familiarity moves neural state boundaries earlier in time, making the optimal delay of familiar subjects shorter. Indeed, the median of the lower-left quadrant is equal to -0.008 s. To statistically test whether this number is significantly below 0, we permuted the subject by subject matrix. This was done by shuffling the order of the subjects 10.000 times to create a null distribution, while labeling the first 6 subjects as “Novel” and the last 5 subjects as “Familiar”, as was the case for the original non-permuted data. In the final analysis, the boundaries of Familiar subjects were not earlier in time than those of Novel subjects ($p = 0.6331$). We therefore conclude that the Novel/Familiar distinction between subjects did not affect boundary timing, and thus that the conclusions of our main analyses still hold.

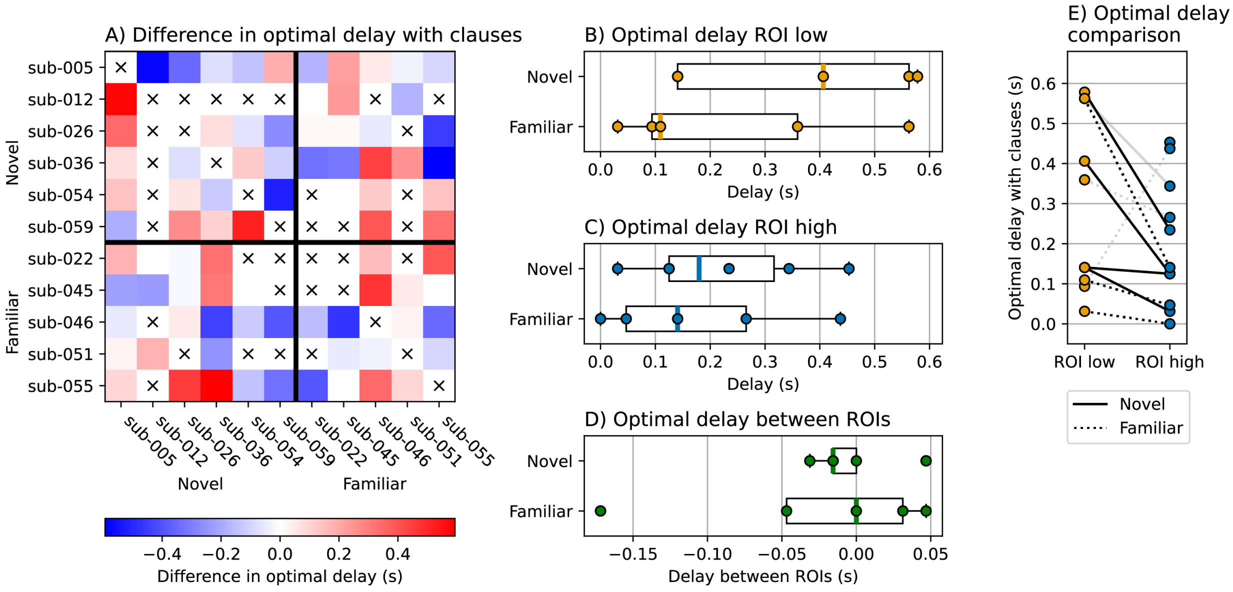

Fig. S5. Stimulus familiarity did not affect timing results. A) Difference in optimal delay with clauses between subjects in the Novel and Familiar groups. A negative number (blue) means that the optimal delay with clauses was shorter for the subject on the y-axis than for the subject on the x-axis. A cross indicates that the subjects could not be compared because of having too few electrodes in common. B and C) Optimal delay with clauses in the low-level and the high-level ROIs. These are the same data as in Figure 3A and B, now divided up into the Novel and the Familiar groups. D) Optimal delay between ROIs. These are the same data as in Figure 4, but divided up into the Novel and the Familiar groups. E) Optimal delays with clauses per subject. Each line is one subject. Gray lines are subjects with a Gaussian match at or below chance level in at least one ROI. These are the same data as in Figure 3C, but divided up into the Novel and the Familiar groups.

If an anticipation effect had been present, it could have affected the results of our main analysis regarding optimal delays with speech and between ROIs (Figures 3 and 4 in the main text). These results are presented again in Figure S5B, C, D and E, but now with Novel and Familiar subjects separated. In line with the absence of an anticipation effect, the optimal delays are not substantially different between Novel and Familiar subjects.

References

Fan, L., Li, H., Zhuo, J., Zhang, Y., Wang, J., Chen, L., Yang, Z., Chu, C., Xie, S., Laird, A. R., et al. (2016). The human brainnetome atlas: a new brain atlas based on connectional architecture. *Cerebral cortex*, 26(8):3508–3526.

Lee, C. S., Aly, M., and Baldassano, C. (2021). Anticipation of temporally structured events in the brain. *Elife*, 10:e64972.
